# Limited Susceptibility of Chickens, Turkeys, and Mice to Pandemic (H1N1) 2009 Virus

**DOI:** 10.3201/eid1604.091491

**Published:** 2010-04

**Authors:** Donata Kalthoff, Christian Grund, Timm C. Harder, Elke Lange, Thomas W. Vahlenkamp, Thomas C. Mettenleiter, Martin Beer

**Affiliations:** Friedrich-Loeffler-Institut, Greifswald-Insel Riems, Germany

**Keywords:** Swine influenza, pandemic (H1N1) 2009, pathogenicity, transmission, chickens, turkeys, mice, influenza, viruses, dispatch

## Abstract

To determine susceptibility of chickens, turkeys, and mice to pandemic (H1N1) 2009 virus, we conducted contact exposure and inoculation experiments. We demonstrated that chickens were refractory to infection. However, oculo-oronasally inoculated turkeys and intranasally inoculated mice seroconverted without clinical signs of infection.

The current outbreak of pandemic (H1N1) 2009 continues to expand in humans, with occasional spillover into domestic pigs. Pandemic (H1N1) 2009 virus causes only mild disease compared with pandemic influenza viruses from the 20th century. However, this characteristic might change if pandemic (H1N1) 2009 viruses acquire virulence markers by reassorting with influenza viruses that cause severe disease in humans, such as highly pathogenic avian influenza viruses (HPAIVs) of the H5N1 subtype. Such reassortment might occur in humans but appears more likely in so-called mixing vessels. Pigs, which had been described as potential mixing vessels (*1*), are highly susceptible to infection with pandemic (H1N1) 2009 virus (*2**,**3*). In addition, pigs have been infected subclinically by HPAIV (H5N1) in countries to which HPAIV (H5N1) is endemic (*4*). However, whether poultry can be infected with pandemic (H1N1) 2009 virus is not well understood. Therefore, we determined the susceptibility of chickens, turkeys, and mice to pandemic (H1N1) 2009 virus.

## The Study

All animal experiments were reviewed and approved by the responsible state ethics committee (LALLF M-V/TSD/7221.3–2.1.-014/09). Five chickens (12 weeks of age, specific pathogen–free [spf]) were inoculated oculo-oronasally by dripping a 10^6^ 50% tissue culture infectious dose (TCID_50_) of virus A/Regensburg/D6/09/H1N1 on the cornea, nares, and oropharynx. Ten chickens (3 months of age, spf) were inoculated intravenously with 10^4^ TCID_50_. Five chickens (15 weeks of age, spf) were housed (permanent contact behind bars and daily direct contact for 30 min) with pigs infected with pandemic (H1N1) 2009 virus (*3*).

A second transmission experiment was performed with the same contact exposure experimental setup, which included 8 infected pigs and 5 turkeys (4 weeks of age). In addition, 28 chickens (1 week of age) and 28 turkeys (1 week of age) were inoculated with 200 μL of virus suspension (10^6^ TCID_50_) directly into the left air sac. Six fattening turkeys (16 weeks of age) from a local flock were inoculated oculo-oronasally with 10^6^ TCID_50_ of A/Regensburg/D6/09 (H1N1) virus. Six fattening turkeys were inoculated oculo-oronasally with 10^8^ TCID_50_ of the more recently isolated pandemic (H1N1) 2009 A/Bayern/74/2009 virus. Cloacal and oropharyngeal swab samples from poultry infected oculo-oronasally, through the air sac, or by contact with pigs were collected daily. Samples were tested by using a real-time reverse transcription–PCR with subtype H1N1–specific primers (http://offlu.net) (Table).

**Table:** Susceptibility of chicken, turkeys, and mice to pandemic (H1N1) 2009 virus*

Animal and dose (virus strain)	No. animals	Route of inoculation	Swab samples	Tissue samples, no. positive/no. tested†	Seroconversion in NP-ELISA (no. positive/no. tested)‡
Chicken	5	Oculo-oronasal	–	NA	–
Chick	28	Intra–air sac	–	Day 2: 2/3; Day 4: 2/3; Day 8: 1/3	–
Chicken	10	Intravenous	NA	NA	–
Chicken	5	Contact with infected pigs	–	NA	–
Turkey, 10^6^ TCID_50_ (A/Regensburg/D6/2009)	6	Oculo-oronasal	–	NA	+ (3/6)
Turkey, 10^8^ TCID_50_ (A/Bayern/74/2009)	6	Oculo-oronasal	+ (respiratory), – (cloacal)	NA	+ (4/6)
Poult	28	Intra–air sac	–	Day 2: 3/3; Day 4: 1/3; Day 6: 1/3	–
Turkey	5	Contact with infected pigs	–	NA	–
Mouse, 10^2^–10^3^ TCID_50_	8	Oculo-oronasal	NA	NA	–
Mouse, 10^4^ TCID_50_	4	Oculo-oronasal	NA	NA	+ (3/4)
Mouse, 10^5^ TCID_50_	4	Oculo-oronasal	NA	NA	+ (4/4)
Mouse, 10^5^ TCID_50_	4	Intraperitoneal	NA	NA	–

*NP, nucleoprotein; –, negative; NA, not applicable; +, positive; TCID_50_, 50% tissue culture infectious dose. †Three juvenile chickens and turkeys inoculated through the air sac were killed on days 0, 2, 4, 6, 8, and 14 posinoculation, and left and right lung lobes were removed for analysis by real-time reverse transcription–PCR. Tissues from the left (inoculation site) lung lobe of 5 chicks and 4 poults killed at the indicated days postinfection were positive for virus RNA with high cycle threshold values (>35), and 1 additional poult was positive for virus RNA with a cycle threshold value of 27 on day 2 postinfection. ‡Seroconversion was verified by testing for antibodies against virus NP by commercially available ELISAs (ELISA Avian Influenza Type A; Pourquier, Montpellier, France, and FlockChek AI MultiS-Screen; IDEXX, Ludwigsburg, Germany).

Additional studies were conducted to determine the 50% lethal dose of strain A/Regensburg/D6/2009 pandemic (H1N1) 2009 virus for mice. Sixteen BALB/c mice (6 weeks of age) were inoculated intranasally with 10^2^–10^5^ TCID_50_/animal (30 μL), and 4 mice were inoculated intraperitoneally with 10^5^ TCID_50_/animal (30 μL).

None of the inoculated animals became ill after infection by any tested route; the intravenous pathogenicity index of the virus for chickens was 0. All swab samples from poultry after contact with infected pigs or from poultry inoculated through the air sac were negative for virus (Table). Virus excretion by pigs was detected (*3*); real-time reverse transcription–PCR cycle threshold values >26 (contact experiment with chickens) or >17 (contact experiment with turkeys). Virus RNA was detected (cycle threshold values 27–39) in swab samples obtained 1–6 days postinoculation from the oropharynx of turkeys inoculated with 10^8^ TCID_50_ of the A/Bayern/74/2009 (H1N1) strain. Small amounts of virus RNA also were detected in the lung and left air sac from a few chicks and poults early after infection through the air sac (Table), which most likely represent residual inoculum. Although contact exposure pigs were infected (*3*), seroconversion was not detected in any of the tested contact exposure poultry species. Poultry inoculated intravenously or through the air sac and chickens inoculated oculo-oronasally were negative for antibodies against influenza A virus nucleoprotein.

Fattening turkeys seroconverted after oculo-oronasal inoculation with 10^6^ TCID_50_ or 10^8^ TCID_50_ (Table). In addition, 7 of 8 mice inoculated with 10^4^ TCID_50_ or 10^5^ TCID_50_ by the oculo-oronasal route seroconverted; 4 mice inoculated with 10^5^ TCID_50_ by the intraperitoneal route showed no detectable antibody response to the virus (Table).

## Conclusions

Our results demonstrate lack of susceptibility of chickens and minor susceptibility of turkeys for infection by pandemic (H1N1) 2009 virus. Transmission of swine influenza viruses to poultry, particularly to turkeys, has been demonstrated (*5*), and experimental infection of chickens with virus subtype H3N2 resulted in a low replication rate, primarily in the alimentary tract (*6*). In contrast, our data indicate that pandemic (H1N1) 2009 virus cannot productively infect chickens at the ages of 1 week (air sac inoculation), 12 weeks (contact exposure, intravenous inoculation), or 15 weeks (oculo-oronasal inoculation), or turkeys at the ages of 1 week (air-sac inoculation) and 4 weeks (contact exposure) because neither virus excretion nor seroconversion was observed during the 10-day observation period. Fattening turkeys from a local flock seroconverted after oculo-oronasal inoculation, but virus RNA was detected in respiratory samples only in turkeys inoculated with a high dose of the A/Bayern/74/2009 (H1N1) virus (Table).

On the basis of our experiments, risk for transmission of pandemic (H1N1) 2009 virus strain to chicken and turkeys and subsequent possible reassortment with other avian influenza viruses should be low. However, we observed a slightly higher susceptibility of older turkeys to high doses of pandemic (H1N1) 2009 virus, which is consistent with recent reports of infected layer turkey flocks in Chile and Canada (*7**,**8*). Analysis of specific virus strains involved in these outbreaks would be useful for confirming these observations and analyzing different strains. Host factors (age, physiologic state, stress levels, and concurrent infections) influencing susceptibility of turkeys should be investigated. Our data, which demonstrate absence of illness in poultry after inoculation with pandemic (H1N1) 2009 virus, are consistent with those of recent experimental infections of poultry, including turkeys (*9*–*11*). Seroconversion was detected only in turkeys by using a hemagglutination-inhibition test in 1 study (*9*), which might indicate higher sensitivity of competitive ELISAs used in our study.

As in other studies (*12**,**13*), characterization of pandemic (H1N1) 2009 virus strains in BALB/c mice showed differences in lethal dose and clinical signs dependent on the virus strain. None of the infected BALB/c mice in our study showed clinical signs. These findings may have resulted from the fact that mice were infected intranasally without anesthesia and with a dose <10^6^ TCID_50_/animal or because of a different phenotype of the virus strain used. However, replication competence of pandemic (H1N1) 2009 virus in mice without prior adaptation was indicated by seroconversion, at least for the higher infectious doses (Table). Intraperitoneal inoculation did not cause development of influenza virus–specific antibodies. This finding cannot be explained by receptor specificity of pandemic (H1N1) 2009 virus because Childs et al. (*14*) showed that representative pandemic (H1N1) 2009 viruses bound not only to most α2–6-linked sialyl moieties, irrespective of the backbone chain length and type, but also to a considerable range of α2–3-linked sialyl residues. Although virus replication may be reduced after intraperitoneal inoculation, development of neutralizing antibodies after inoculation by this route has been reported (*15*).

Our results demonstrate lack of susceptibility and absence of serologic responses of chickens and turkeys to contact infection with an early human-origin isolate of pandemic (H1N1) 2009 virus. However, direct inoculation of fattening turkeys resulted in seroconversion and detection of virus RNA in oropharyngeal swab samples. Generation of new variants and reassortants caused by double infections with other influenza A viruses and pandemic (H1N1) 2009 virus in poultry other than turkeys appears unlikely. However, further adaptation of pandemic (H1N1) virus strains in turkeys cannot be excluded.
